# Hyperactive learning for data-driven interatomic potentials

**DOI:** 10.1038/s41524-023-01104-6

**Published:** 2023-09-13

**Authors:** Cas van der Oord, Matthias Sachs, Dávid Péter Kovács, Christoph Ortner, Gábor Csányi

**Affiliations:** 1 University of Cambridge, Cambridge, CB2 1PZ UK; 2 University of Birmingham, Birmingham, B15 2TT UK; 3 University of British Columbia, Vancouver, BC V6T 1Z2 Canada

**Keywords:** Atomistic models, Computational methods

## Abstract

Data-driven interatomic potentials have emerged as a powerful tool for approximating ab initio potential energy surfaces. The most time-consuming step in creating these interatomic potentials is typically the generation of a suitable training database. To aid this process hyperactive learning (HAL), an accelerated active learning scheme, is presented as a method for rapid automated training database assembly. HAL adds a biasing term to a physically motivated sampler (e.g. molecular dynamics) driving atomic structures towards uncertainty in turn generating unseen or valuable training configurations. The proposed HAL framework is used to develop atomic cluster expansion (ACE) interatomic potentials for the AlSi10 alloy and polyethylene glycol (PEG) polymer starting from roughly a dozen initial configurations. The HAL generated ACE potentials are shown to be able to determine macroscopic properties, such as melting temperature and density, with close to experimental accuracy.

## Introduction

Over the last decade there has been rapid progress in the development of data-driven interatomic potentials, see the review papers^1–6^. Many systems are often too complex to be modelled by an empirical description yet inaccessible to electronic structure methods due to prohibitive computational cost. Richly parametrised data-driven interatomic potentials bridge this gap and are able to successfully describe the underlying chemistry and physics by approximating the potential energy surface (PES) with quantum mechanical accuracy^7–9^. This approximation is done by regressing a high-dimensional model to training data collected from electronic structure calculations.

Over the years many approaches have been explored using a range of different model architectures. These include artificial neural networks (ANN) based on atom centred symmetry functions^10^ and have been used in models such as ANI^11,12^ and DeepMD^5^. Another widely used approach is Gaussian process regression (GPR) implemented in models such as SOAP/GAP^13,14^, FCHL^15^ and sGDML^16^. Linear approximations of the PES have also been introduced initially by using permutation invariant polynomials (PIPs)^17^ and the more recent atomic PIPs variant^18,19^. Other linear models include spectral neighbour analysis potentials^4^ based on the bispectrum^20^, moment tensor potentials^21^ and the atomic cluster expansion (ACE)^22–24^. More recently, message passing neural network (MPNN) architectures have been introduced^25–31^ the most recent of which have been able to outperform any of the previously mentioned models regarding accuracy on benchmarks such as MD17^32^ and ISO17^33^. Central to all of these models is that they are fitted to a training database comprised of configurations *R* labelled with total energy ${{{{\mathcal{E}}}}}_{R}$, forces ${{{{\mathcal{F}}}}}_{R}$ and perhaps virial stress ${{{{\mathcal{V}}}}}_{R}$ observations, obtained from electronic structure calculations. By performing a regression on the training data model predictions *E* of the total energy, and estimates of the respective forces *F*_*i*_ = −∇_*i*_*E* can be determined. Here, the ∇_*i*_ operator denotes the gradient with respect to the position of atom *i*.

Building suitable training databases remains a challenge and the most time-consuming task in developing general data-driven interatomic potentials^34–36^. Databases such as MD17 and ISO17 are typically created by performing molecular dynamics (MD) simulations on the structures of interest and selecting decorrelated configurations along the trajectory. This approach samples the potential energy surface according to its Boltzmann distribution. Once the training database contains sufficient number of configurations, a high-dimensional model may be regressed in order to accurately interpolate its potential energy surface. The interpolation accuracy can be improved by further sampling, albeit with diminishing returns. However, it is by no means clear that the Boltzmann distribution is the optimal measure, or even a “good” measure, from which to draw samples for an ML training database. Indeed, it likely results in severe undersampling of configurations corresponding to defects and transition states, particularly for material systems with high barriers, which nevertheless have a profound effect on material properties and are often the subject of intense study.

A lack of training data in a sub-region can lead to deep unphysical energy minima in trained models, sometimes called “holes”, which are well known to cause catastrophic problems for MD simulations: the trajectory can get trapped in these unphysical minima or even become numerically unstable for normal step sizes. A natural strategy to prevent such problems is active learning (AL): the simulation is augmented with a stopping criterion aimed at detecting when the model encounters a configuration for which the prediction is unreliable. Intuitively, one can think of such configurations as being “far” from the training set. When this situation occurs, a ground-truth evaluation is triggered, the training database extended, and the model refitted to the enlarged database. In the context of data-driven interatomic potentials, this approach was successfully employed by the linear moment tensor potentials^37,38^ and the Gaussian process (GP) based methods FLARE^39,40^ and GAP^41^ which both use site energy uncertainty arising from the GP to formulate a stopping criterion in order to detect unreliable predictions during simulations.

The key contribution of this work is the introduction of the hyperactive learning (HAL) framework. Rather than relying on normal MD to sample the potential energy and wait until an unreliable prediction appears (which may take a very long time once the model is decent), we continually bias the MD simulation towards regions of high uncertainty. By balancing the physical MD driving force with such a bias, we accelerate the discovery of unreliably predicted configurations but retain the overall focus on low energy configurations carrying large contributions to the partition function. This proposed framework is reminiscent of the exploration-exploitation trade-off originating from Bayesian optimisation (BO), a technique used to efficiently optimise a computationally expensive “black box” function. BO has been shown to yield state-of-the-art results for optimisation problems while simultaneously minimising incurred computational costs by requiring fewer ground-truth evaluations^42^. In the wider community BO is seen as a type of AL, and so is the proposed HAL framework in this work. The novelty of this work is combining MD with BO to accelerate the development of data-driven interatomic potentials.

BO has been applied to atomistic systems previously in global structure search^43–46^ where the PES is optimised to find stable structures. Other previous work balancing exploration and exploitation in data-driven interatomic potentials is also closely related, where configurations were generated by balancing high uncertainty and high-likelihood (or rather low-energy)^47^. Here the PES was explored by perturbing geometries while monitoring uncertainty rather than explicitly running MD. Note that upon the completion of this work, we discovered a closely related work that also uses uncertainty-biased MD^48^. The two studies were performed independently, and appeared on preprint servers near-simultaneously.

In BO, an acquisition function balances exploration and exploitation, controlled by a biasing parameter. In our hyperactive learning framework, the HAL potential energy surface *E*_HAL_:

1 $${E}_{{{{\rm{HAL}}}}}:= E-\tau \sigma$$

takes on a similar role. Here, *E* is the predicted potential energy and *σ* is an uncertainty measure, which in this work is set to be the standard deviation of predicted total energy. The parameter *τ*, referred to as the biasing strength, controls the exploration of unseen parts of the PES and needs to be carefully tuned in order for the HAL-MD trajectory to remain energetically sensible. This is achieved by the introduction of an on-the-fly auto-tuning scheme using a relative biasing parameter τ_*r*_ (see § “Methods” for details). The addition of a biasing potential has a long history in the study of rare events and free energy computations, using adaptive biasing strategies such as meta-dynamics^49,50^, umbrella sampling^51,52^, and similar methods (e.g. refs. ^53,54^). While the biasing force in these methods is implicitly specified by the choice of a collective variable, the direction of the biasing force in HAL is towards increasing uncertainty corresponding to regions of configuration space not accurately described by the training data. Viewing HAL as an adaptive-biasing technique also contrasts it against more aggressive AL approaches that explore configuration space via thermostated MD at high temperature. In the latter case all degrees of freedom are indiscriminately accelerated. In the absence of strong energetic barriers this drastically increases the size of the sampled configurational space rendering an exhaustive exploration of physically relevant configurations infeasible. In contrast, HAL only accelerates the degrees of freedom in the direction of increasing uncertainty. Intuitively, one may expect that this keeps the size of sampled configurational space constrained and exploration effective.

Choosing *σ* to be the predicted energy’s standard deviation makes *E*_HAL_ coincide exactly with the lower confidence bound (LCB), which is a commonly used acquisition function in BO. In particular, it has previously been used to optimise the potential energy surface^43^ rather than to sample the corresponding statistical ensembles as performed in this work. From both a theoretical and modelling perspective other versions of HAL are of high interest. For example, we expect that using the relative force uncertainties that we introduce below as biasing potentials, would result in a more targeted biasing that is consistent with the proposed stopping criterion presented in Eq. (6). However, since such a formulation of HAL would require the evaluation of higher order derivatives of the predicted energy, we leave this to future work.

We make the general HAL concept concrete in the context of the ACE “machine learning potential” framework^22,23^, however, the methods we propose can be directly applied to any linear models and Gaussian process type models, and are in principle also extendable to any other ML potential that comes with an uncertainty measure, including deep neural network models. Different methods of setting up such ensembles or committees exist for linear, GP or NN frameworks, such as dropout^55^, or bootstrapping^56^. In the context of Bayesian models, ensembles can be obtained as Monte Carlo samples from the corresponding posterior distribution. More specifically, considering linear ACE, the site (or atomic) energy is expressed as follows:

2 $${E}_{i}={{{\bf{c}}}}\cdot {{{{\boldsymbol{B}}}}}_{i}.\quad$$

and the total energy *E* is defined as *E* = ∑_*i*_*E*_*i*_ = **c** ⋅ ${\boldsymbol{B}}$ where ${\boldsymbol{B}}$ = ∑_*i*_${\boldsymbol{B}}_i$ is the linear ACE basis. Due to this linearity, implementing a Bayesian model formulation is particularly straightforward allowing for efficient and analytical uncertainty estimation as described in § “Bayesian ridge regression (BRR)”. Assuming an isotropic Gaussian prior on the model parameters and Gaussian independent and identically distributed (i.i.d) noise on observations, yields an explicit definition of the standard deviation of the posterior-predictive distribution:

3 $$\sigma =\sqrt{\frac{1}{\lambda }+{{{{\boldsymbol{B}}}}}^{T}{{{\mathbf{\Sigma }}}}{{{\boldsymbol{B}}}}},$$

which has energy units, in correspondence with Eq. (1). Here, the covariance matrix **Σ** is defined as:

4 $$\mathop{\Sigma}\nolimits^{-1}=\alpha {{{\bf{I}}}}+\lambda {{{{\mathbf{\Psi }}}}}^{T}{{{\mathbf{\Psi }}}}.$$

and *α*, *λ* are hyperparameters whose treatment are detailed in the Methods section, and **Ψ** is the design matrix of the linear regression problem.

The evaluation of *σ* in Eq. (3) is computationally expensive for a large basis ${\boldsymbol{B}}$; scaling as $O({N}_{{{{\rm{basis}}}}}^{2})$. To improve computational efficiency, *σ* can be approximated by a committee of *K* potentials parameterised by an ensemble of parameters ${\{{{{{\bf{c}}}}}^{k}\}}_{k = 1}^{K}$ that are obtained by sampling from the posterior distribution *π*(**c**) (see Eq. (25) for further details). This gives rise to *K* committee energy predictions, *E*^*k*^ = **c**^*k*^ ⋅ ${\boldsymbol{B}}$, resulting in:

5 $$\tilde{\sigma }=\sqrt{\frac{1}{\lambda }+\frac{1}{K}\mathop{\sum }\limits_{k=1}^{K}{({E}^{k}-\bar{E})}^{2}},$$

where $\bar{E}=\bar{{{{\bf{c}}}}}\cdot {{{\bf{B}}}}$ with $\bar{{{{\bf{c}}}}}$ being the mean of the posterior distribution. We provide the explicit form of $\bar{{{{\bf{c}}}}}$ in Eq. (25). The expression for $\tilde{\sigma }$ is computationally efficient to evaluate, requiring a single basis evaluation ${\boldsymbol{B}}$ followed by *K* + 1 dot-products with the mean $\bar{{{{\bf{c}}}}}$ and committee parameterisations **c**^*k*^.

Having introduced *E*_HAL_, it remains to specify an uncertainty measure, or stopping criterion, to terminate the dynamics, identifying new training configurations and extending the training database (as used in AL). To that end, we introduce a relative force uncertainty, *f*_*i*_, which is attractive from a modelling perspective: For instance, liquid and phonon property predictions require vastly different absolute force accuracy but rather similar relative force accuracy, typically on the order of 3–10%. Given the committee forces ${F}_{i}^{k}=-{\nabla }_{i}{E}^{k}={{{{\bf{c}}}}}^{k}\cdot {\nabla }_{i}{{{\bf{B}}}}$, we define:

6 $${f}_{i}=\frac{\frac{1}{K}\mathop{\sum }\nolimits_{k = 1}^{K}\parallel {F}_{i}^{k}-{\bar{F}}_{i}\parallel }{\parallel {\bar{F}}_{i}\parallel +\varepsilon },$$

where ${\bar{F}}_{i}$ is force prediction as predicted by the mean parameters $\bar{{{{\bf{c}}}}}$. Further, *ε* is a regularising constant to prevent divergence of the fraction. This parameter is specified by the user and should be chosen to be smaller than the typical force magnitude observed during dynamics. In practice it is found that 0.2–0.4 eV/Å generally yields good behaviour.

During HAL simulations, *f*_*i*_ provides a computationally efficient means to detect emerging local (relative force) uncertainties and is used to trigger new ab initio calculations once it exceeds a predefined tolerance:

7 $$\mathop{\max }\limits_{i}\,{f}_{i}\, > \,{f}^{{{{\rm{tol}}}}}.$$

The specification of *f*^tol^ is both training data and model specific, and requires careful tuning to achieve good performance. Too small *f*^tol^ keeps triggering unnecessary ab initio calculations, whereas a too large value leads to generation of unphysical high energy configurations. To avoid manual tuning and aid generality, we normalise *f*_*i*_ onto [0, 1] through the application of the softmax function $s({f}_{i})=\exp ({f}_{i})/{\sum }_{i}\exp ({f}_{i})$, and redefine the stopping criterion as:

8 $$\mathop{\max }\limits_{i}\,s({f}_{i})\, > \,{s}^{{{{\rm{tol}}}}}.$$

This setup is chosen to mimic a probabilistic classifier whereby a binary decision is made between two options: triggering a QM calculation or continue HAL dynamics. A default tolerance of *s*^tol^ = 0.5 is used as it correspond to the decision boundary between the two options.

**Fig. 1:**
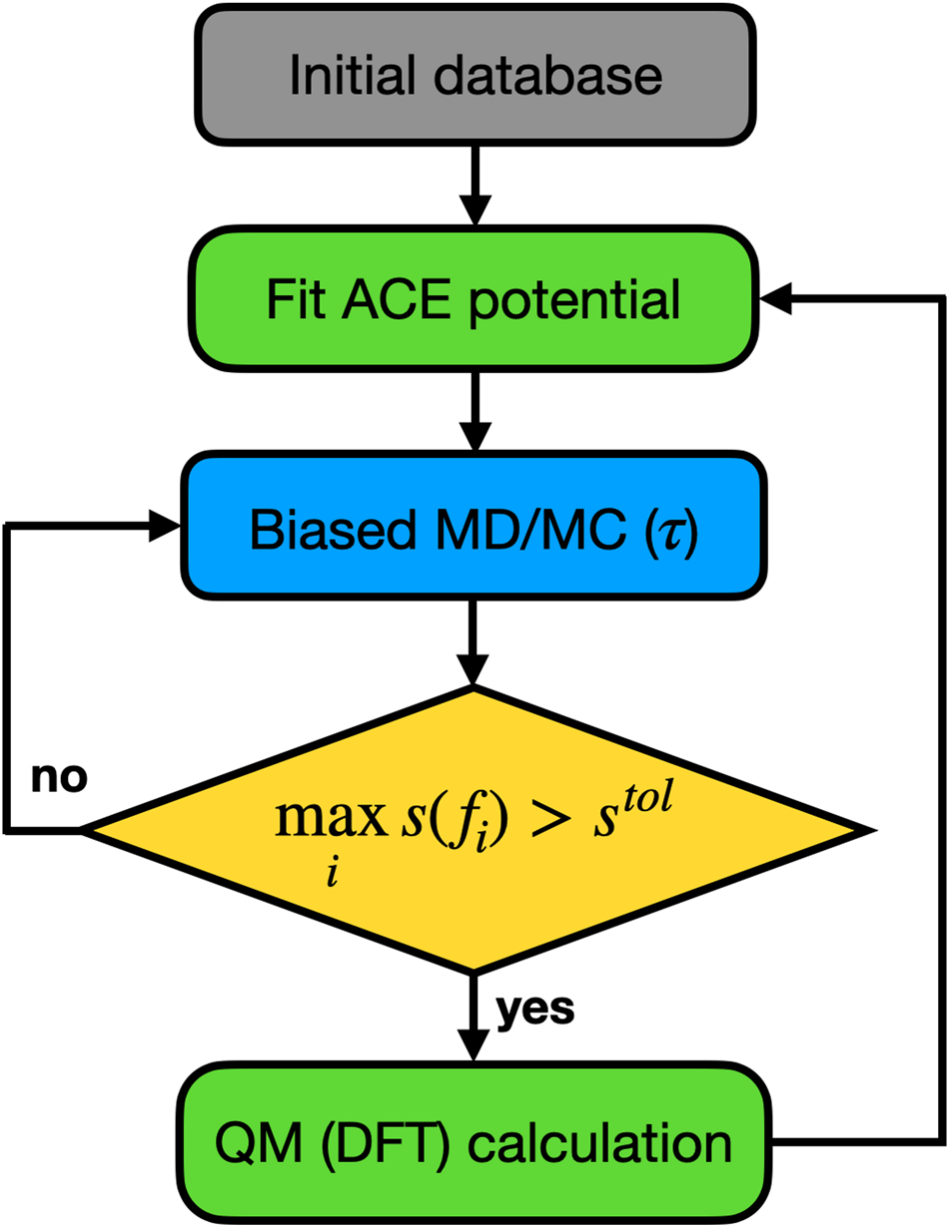
Schematic overview of the HAL framework performing biased AL controlled by a biasing parameter *τ*. The softmax normalised relative force uncertainty *s*(*f*_*i*_) is used as a stopping criterion to trigger DFT calculations.

The main purpose of this work is to present an accelerated AL scheme for generating data-driven interatomic potentials, and showcase it by determining alloy melting temperature and polymer density with close to experimental accuracy. Using an initial database that comprises a few atomistic configurations, the HAL procedure is started by biasing dynamics towards uncertainty while running MD (and optionally Monte Carlo (MC), for e.g. volume changes or atom swaps). If the uncertainty exceeds a predefined (AL) uncertainty tolerance, or stopping criterion (*s*^tol^), during the biased dynamics, a DFT calculation is triggered. This newly labelled configuration is added to the training database and the ML model is refitted. Then the next HAL iteration commences using the newly fitted potential. The HAL scheme is illustrated in Fig. 1 where *τ* corresponds to the biasing parameter in Eq. (1). This parameter requires careful tuning and an on-the-fly adaptive scheme controlled by a relative biasing parameter *τ*_*r*_ is discussed in § “Methods”.

The remainder of this article is organised as follows. In the § “Results and discussion” we first demonstrate the suitability of the relative force error measure *f*_*i*_ as a selection criterion in an AL framework by evaluating its correlation with the true relative force error and by using it to sequentially re-assemble a much-reduced diamond structure silicon database (section “Results and discussion”). In sections “AlSi10” and “Polyethylene glycol (PEG)” we show how the HAL framework can be used to build training databases from scratch in the case of an alloy (AlSi10) and polymer (polyethylene glycol or PEG), respectively. The assembled training databases are shown to contain sufficient information to fit ACE potentials that enable stable simulation of MD trajectories on long time scales and can accurately predict macroscopic properties such as the melting temperature of AlSi10 and the density of PEG. Section “Methods” describes the HAL scheme with § “Hyperactive learning (HAL)”) describing in detail the Monte Carlo estimate of the HAL biasing force and the adaptive on-the-fly auto-tuning scheme for the determination of the relative biasing parameter *τ*_*r*_. A brief recap of ACE is also provided in § “Atomic cluster expansion (ACE)” as well as detailed description of the Bayesian regression methods that we use to obtain the uncertainty measures in HAL (§ “(Bayesian) Linear regression” to “Posterior predictive distribution”).

## Results and discussion

### Silicon database filtering

Before illustrating the HAL algorithm itself, we first demonstrate the ability of the relative force uncertainty estimate *f*_*i*_ in Eq. (6) to detect true relative force errors. To that end, we will use this estimator to significantly reduce a large training set while maintaining accurate model properties relative to the DFT reference. The database we use for this demonstration was originally developed for a silicon GAP model^35^ and covers a wide range of structures ranging from bulk crystals in various phases, amorphous, liquid and vacancy configurations. The filtering process builds a reduced database by starting from a single configuration and selecting configurations containing the maximum *f*_*i*_ from the remaining test configurations. Iterating this process accelerates the learning rate and rapidly converges model properties with respect to the DFT reference. The models trained are linear ACE models that consist of basis functions up to correlation order *ν* = 3, polynomial degree 20, outer cutoff set to 5.5 Å and inner cutoff set to the closest interatomic distance in the training database. An auxiliary pair potential basis was used using polynomial degree 3, outer cutoff 7.0 Å and no inner cutoff. The weights for the energy *w*_*E*_, forces *w*_*F*_ and virials *w*_*V*_, which are described in detail in the “Methods” section, were set to 5.0/1.0/1.0. The size of the committees used to determine *f*_*i*_ was *K* = 32.

**Fig. 2:**
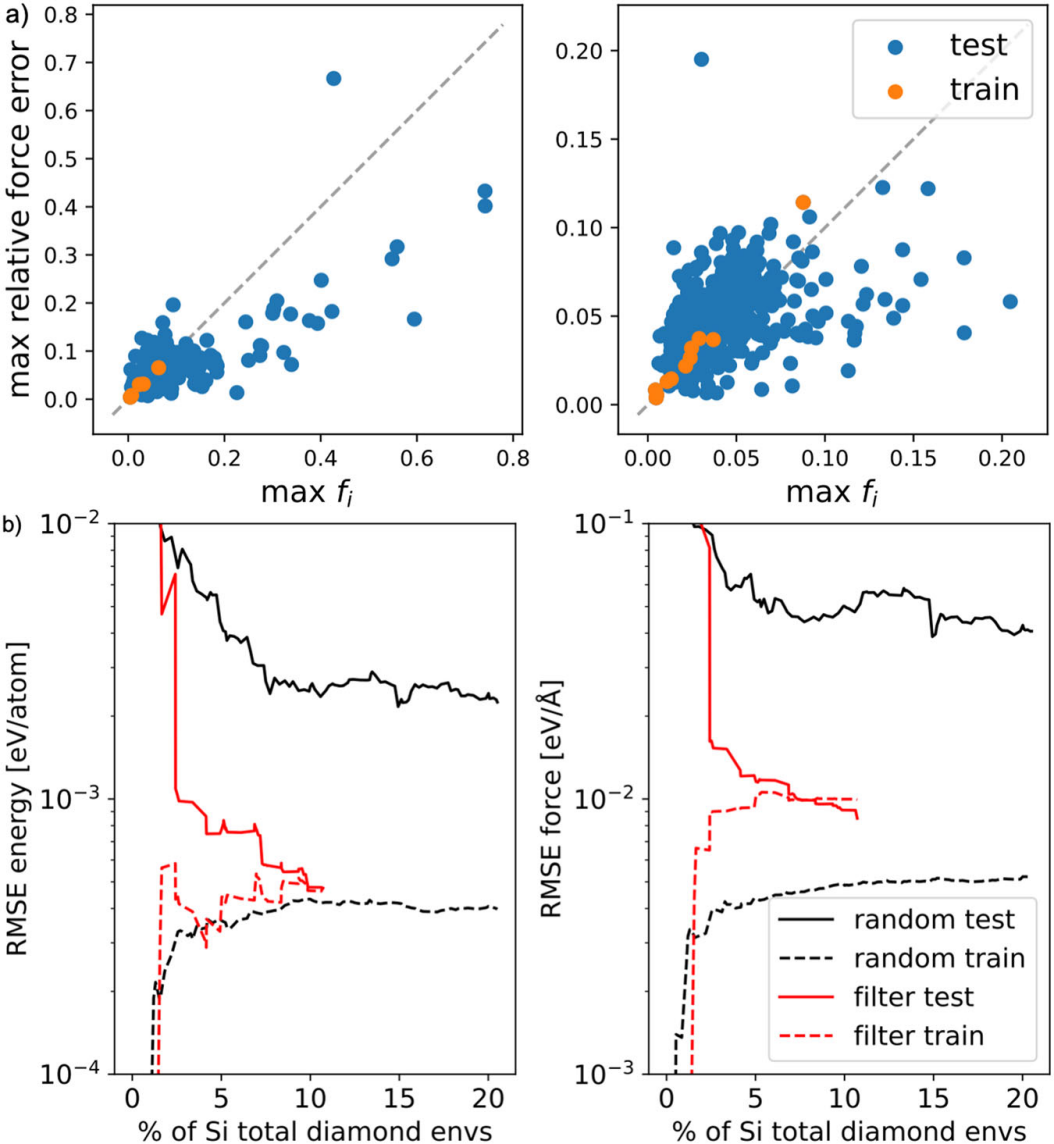
Benchmarking relative force uncertainty *f*_*i*_ for filtering silicon diamond database. **a** Maximum relative force error estimate $\max {f}_{i}$ vs. error correlation plots for silicon diamond containing 4 (left) and 10 (right) training configurations. **b** Learning rate benchmark comparing filtering and random selection for silicon diamond energy (left) and forces (right).

Prior to training database reduction the ability of the relative force uncertainty estimate *f*_*i*_ to predict relative force error is investigated. Fig. 2a compares the maximum relative force error in a configuration against the maximum of *f*_*i*_ for two different training databases, containing 4 and 10 silicon diamond configurations respectively. The test configurations are the remaining configurations contained in the 489 silicon diamond configurations that comprise of the entire silicon database (which in total contains 16,708 atomic site neighbour environments). The regularising constant *ε* in Eq. (6) was set to the mean force magnitude as predicted by the mean parameterisation. Both figures show good correlation between maximum relative force error and $\max {f}_{i}$, therefore making it a suitable criterion to be monitored during (H)AL strategies.

By leveraging the correlation of *f*_*i*_ with true relative force error the existing silicon diamond database can be reduced by iteratively selecting configurations containing the largest relative force uncertainty as part of a greedy algorithms strategy. To demonstrate this, a randomly selected single configuration from the 489 silicon diamond configurations of the silicon database was fitted. Next, *f*_*i*_ was determined over the remaining configurations and the configuration containing the largest $\max {f}_{i}$ was added to the training database. This process was repeated. The train and test error for both the energies and forces during this silicon diamond filtering procedure are shown in Fig. 2b. It is benchmarked against performing random selection whereby, starting from the same initial configuration, configurations were chosen at random from the pool of remaining configurations of the training database. The result indicates that *f*_*i*_ accurately detects configurations with large errors and manages to accelerate the learning rate significantly relative to random selection. Good generalisation between training and test errors is achieved by using around 5% of the total environment contained in the original silicon diamond database.

The significant acceleration of the learning rate shown in Fig. 2b shows that generalisation between train and test error is rapidly achieved, in turn suggesting that property convergence is accelerated too. This is investigated by comparing macroscopic properties of the DFT reference with predictions of the ACE models that were fitted as part of the filtering process. These macroscopic properties include elastic constants, energy volume curves, phonon spectrum and thermal properties for bulk silicon diamond. Results are reported for the ACE models that were fitted to 9 configurations (424 environments), 13 configurations (460 environments) and 17 configurations (608 environments), which, respectively, amount to ~3, 4 and 5% of silicon diamond environments contained in the original database.

**Fig. 3:**
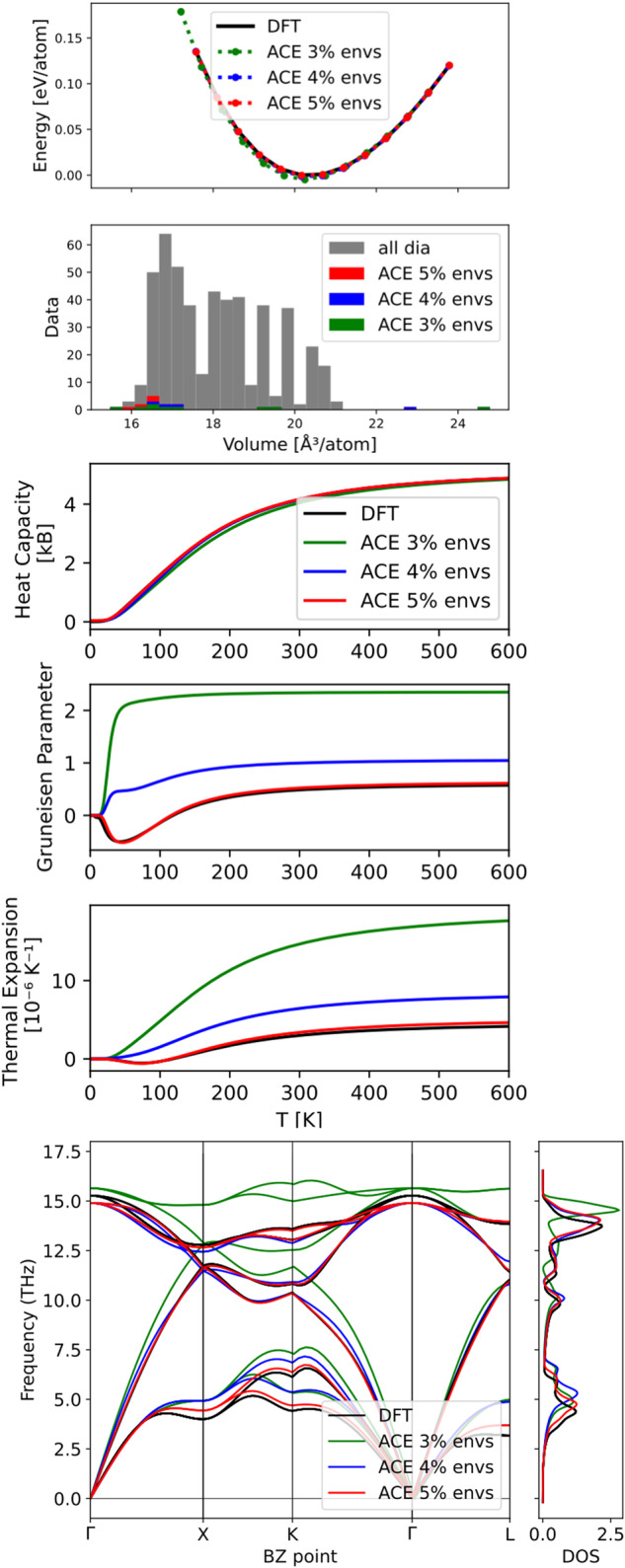
Property convergence for filtered silicon diamond ACE potentials. Properties included are energy versus volume (top), thermal properties (middle) and phonon spectrum (bottom).

Figure 3 demonstrates that property convergence for the energy volume curves, phonon spectrum and thermal properties are rapidly achieved by fitting to a fraction of the original database. Most notably the negative thermal expansion is reproduced, as observed experimentally^57^ and by DFT. This property is particularly challenging to get right and many empirical models fail as shown in the original silicon GAP work^35^. Fitting to 5% of the original database reaches sufficient accuracy to describe all properties with good accuracy with respect to the DFT reference. This is again confirmed by elastic constants as predicted by the respective models as shown in Table 1. The convergence of the phonon spectrum in Fig. 3 is particularly noteworthy as relative errors on the order of a few percent on small forces ~0.01 eV/Å are typically required to accurately recover the phonon spectrum. The fact that such small relative force errors are achieved while fitting on very few data points is a direct consequence of the design of the filter criterion or uncertainty measure *f*_*i*_.

**Table 1:** Convergence of the elastic moduli (GPa) of the filtered ACE models relative to the CASTEP DFT reference.

	*B*	*c*_11_	*c*_12_	*c*_44_
ACE 3% envs	98.2	188.1	53.3	79.7
ACE 4% envs	84.2	159.8	46.4	75.7
ACE 5% envs	82.5	148.7	49.3	73.7
DFT	82.6	147.2	50.3	73.1

### AlSi10

This section outlines the general HAL protocol for building training databases for alloys and demonstrates how an AlSi10 linear ACE model is built from scratch in an automated fashion. By using the relative force error estimate *f*_*i*_ previously discussed as a stopping criterion to trigger ab initio evaluations it will be shown how an ACE model is created for AlSi10 using HAL. The ACE models used in this section contained basis functions up to correlation order *ν* = 2 and polynomial degree 13 as well as an outer cutoff 5.5 Å. The ACE inner cutoff was set to 1.5 Å during the HAL stage of collecting data and moved towards the closest interatomic distance once all training data had been generated. An auxiliary pair potential *V*_2_ added to aid stability also added to the basis including functions up to polynomial degree 13 and an outer cutoff of 6.0 Å. The weights for the energy *w*_*E*_, forces *w*_*F*_ and virials *w*_*V*_ were set to 15.0, 1.0, 1.0, respectively.

The HAL procedure of building ACE models for alloys starts by creating set of a random crystal structures manually, from which a random alloy and liquid alloy training database are built in an iterative fashion. Once sufficient data for both phases has been collected, the HAL solid and liquid databases are afterwards combined in order to create a model that accurately describes both phases. The first step in the HAL protocol is the creation of a set of small initial random alloy database, which was formed of 32-atom FCC lattice configurations populated with 29 Al and 3 Si atoms, equivalent to 9.7 weight percent Si. This initial random alloy starting database contained ten configurations with lattice constants ranging from 3.80 Å to 4.04 Å and was evaluated using CASTEP^58^ DFT. The main parameters were as follows: plane-wave cutoff 300 eV, kpoint spacing 0.04 Å^−1^, 0.1 eV electronic smearing, Pulay density mixing scheme and finite basis correction.

An adaptive biasing parameter *τ*_r_ = 0.05 was chosen (for explicit definition see “Methods” section) and the temperature set to *T*_solid_ = 800 K in order to build the random solid alloy database starting from the 10 initial structures previously described. Besides running biased dynamics, we performed cell volume changes (by adding Gaussian noise to cell vectors) and atom swapping using Monte Carlo (MC) steps during the simulation in order to assist exploration of unseen configurations. These MC steps were accepted or rejected according to the Metropolis-Hastings algorithm^59^.

**Fig. 4:**
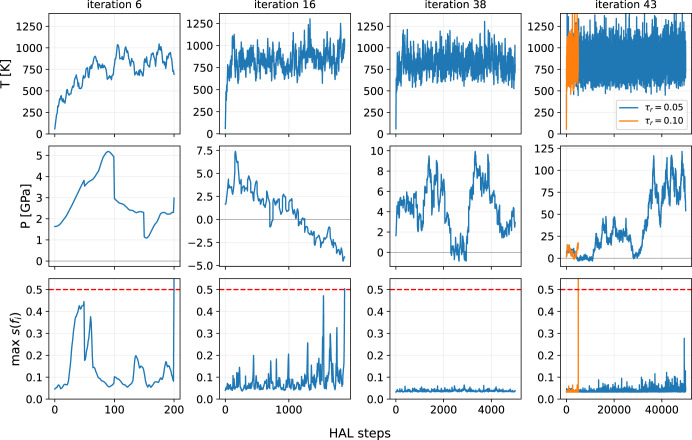
HAL dynamics for several iterations for the AlSi10 random alloy showing maximum softmax normalised relative force error estimate $\max s({f}_{i})$, temperature and pressure. DFT calculations are triggered if the tolerance *s*^tol^ = 0.5 in red is reached. Pressure fluctuations are due to swap/volume MC steps on HAL potential energy surface *E*_HAL_.

During HAL dynamics the softmax normalised relative force estimate *s*(*f*_*i*_) is evaluated and a ground-truth evaluation triggered once a predefined tolerance of *s*^tol^ = 0.5 on any of the atoms is met. A total of 42 HAL configurations were sampled as the HAL dynamics at this stage was stable reliably for 5000 steps. The pressure *P*, temperature *T* and $\mathop{\max }\nolimits_{i}s({f}_{i})$ are shown in Fig. 4 for four exemplary iterations with the first three being included in the training database, e.g. below or equal to iteration 42. The strong oscillations in the pressure *P* are due to the volume and element swapping MC steps being accepted. Finally, as demonstrated in the case of the 43th HAL iteration that increasing the biasing strength to *τ*_r_ = 0.10 results in a drastic acceleration (by a factor of 10) in the discovery of configurations with large relative force error.

Next, HAL was employed to assemble a database of liquid random alloys. HAL trajectories were initialised at configurations sampled by cycling through the training database of random solid alloys obtained in the previous HAL run. HAL trajectories were simulated using a Langevin thermostat targeting a temperature regime of *T*_liquid_ = 3000 K, and a proportional control barostat targeting pressure level of 0.1 GPa. No volume or swap MC steps were performed. After generating 46 liquid alloy configurations using HAL, the HAL dynamics were reliably stable for 5000 steps and the database assembly for this temperature regime was terminated.

Finally, the 42 HAL generated random alloy configurations and 46 HAL generated liquid configurations were combined to form a training database. This training database was used to fit linear ACE models for AlSi10 using Automatic relevance determination (ARD); see section “Automatic relevance determination (ARD)” for details. We considered various thresholds ${\alpha }^{{\prime} }$ for the pruning of model parameters. The performance of the pruned models in terms of computational speed, training and test errors, are shown in Table 2. The test set used to compute test error consisted of 14 solid and 14 liquid configurations. These configurations were obtained by sampling from the corresponding temperature and pressure regimes by continuing the HAL runs. Increasing ${\alpha }^{{\prime} }$ lowers the relevance criterion for the linear ACE basis functions in turn decreasing sparsity. A clear trade-off between sparsity and training error can be seen in Table 2 which also includes model evaluation performance and fitting times. Increasing ${\alpha }^{{\prime} }$ not only decreases training error but also test error up to ${\alpha }^{{\prime} }=300{\rm{k}}$ for which the test error increases, a sign of overfitting. Due to the relatively small training database size the computing time to fit the models remains low, around a minute or less using 8 threads on Intel(R) Xeon(R) Gold 5218 CPU. Performance testing was done using LAMMPs and the PACE evaluator^60^ using Intel(R) Xeon(R) Gold 6142F. The performance tests illustrate scaling trends across different sized cells and cores used for simulation.

**Table 2:** Train/test error splits for HAL generated AlSi10 database for varying ARD tolerance ${\alpha }^{{\prime} }$.

${\alpha }^{{\prime} }$	*N*_basis_	Training error		Test error			Evaluation time								Fit time
						*N*_atoms_:	32		14,336		32		14,336		
		*E*	*F*	*E*	*F*	${N}_{{{{\rm{cores}}}}}$:	1	32	32	448	1	32	32	448	
1k	38	7.693	0.135	8.006	0.147		62	214	72	97	43	403	3	28	2
10k	116	4.199	0.095	6.229	0.104		87	270	100	110	31	319	2	24	3
80k	295	2.401	0.080	5.131	0.089		91	278	105	118	30	310	2	23	17
300k	621	1.869	0.074	5.188	0.095		96	300	115	125	28	287	2	22	63
		(meV/at)	(eV/Å)	(meV/at)	(eV/Å)		(core-μs/atom)				(10^6^ step/day)				(s)

Larger ARD tolerance ${\alpha }^{{\prime} }$ includes more basis functions, increases accuracy but leads to worse performance and fitting time. Performance timings for 32 and 14,336 atom (8,8,7 supercell) sized cells are shown for various core counts. A timestep of 1 fs was used such that 10^6^ step/day is equivalent to ns/day. These performance timings are for illustrative purposes and do not represent a full computational scaling benchmark, which would need to separately address strong and weak scaling.

**Fig. 5:**
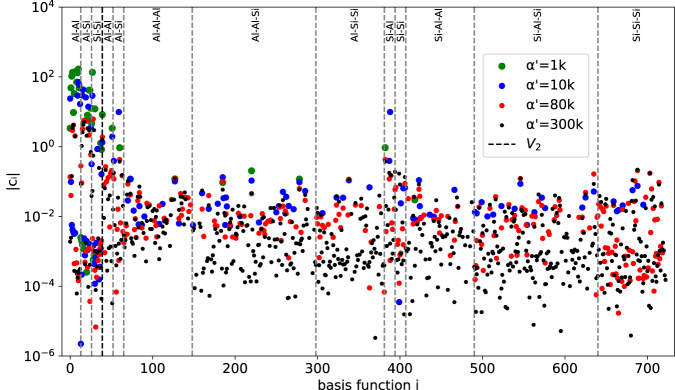
Coefficient magnitude ∣**c**_*i*_∣ for the 723 basis functions grouped per correlation order and element interaction for various ARD tolerances ${\alpha }^{{\prime} }$. Large coefficients are assigned to pair interactions, partly captured by the auxiliary pair potential *V*_2_, as most of the binding energy is contained in these interactions.

Further analysis of the ARD fitted models was done by examining the absolute value of the coefficients ∣**c**_*i*_∣. Basis functions whose estimated prior precision is below the predefined threshold are pruned away as can be seen in Fig. 5. Large coefficients are given to the pair interactions described by the auxiliary basis *V*_2_ and two-body components of the ACE basis for all models, which is intuitive as most binding energy is stored in these pair interactions. Increasing ${\alpha }^{{\prime} }$ results in more (less relevant) basis functions being included with relatively smaller coefficients. For ${\alpha }^{{\prime} }=300k$ many of these low relevance coefficients of around 10^−4^ are included in the fit indicating a degree of overfitting—as confirmed by the test set error increase in Table 2.

**Fig. 6:**
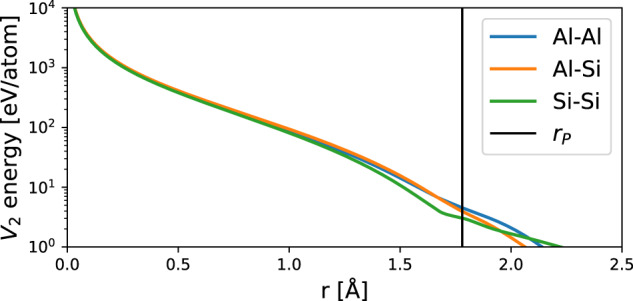
Pair interaction energy at close approach. Core repulsion is used to stabilise the ACE potential for gas-like configurations explored during NS.

Next, the melting temperature for each of the previously ARD fitted AlSi10 ACE models is determined. This was done using Nested Sampling (NS) which approximates the partition function of an atomic system by exploring the potential energy surface over decreasing energy (or enthalpy) levels, in turn determining the cumulative density of states^61,62^. NS expresses the partition function in term of enthalpy *H* for *N* atoms given inverse temperature *β*, momenta **p** and positions **q** as follows:

9 $$\begin{array}{lll}\Delta (N,\beta ,P)\,=\,\displaystyle\int{e}^{-\beta H({{{\bf{q}}}},{{{\bf{p}}}})}d{{{\bf{q}}}}d{{{\bf{p}}}}\\ \qquad\qquad\quad \approx \,\frac{\beta P}{N!{h}^{3N}}\mathop{\sum}\limits_{i}{w}_{i}{e}^{-\beta {H}_{i}}, \end{array}$$

where the algorithm explores phase space volumes *H*_*i*_ of the PES using a top-down approach, i.e. ideal gas to ground structure. From this expression the heat capacity at constant pressure *C*_*P*_ can be determined:

10 $${C}_{P}=-\left(\frac{\partial }{\partial T}\frac{\partial \Delta (N,P,\beta )}{\partial \beta }\right),$$

which exhibits a signature peak at a first order phase transition, such as melting. Extensive previous work has shown that NS is a highly automated, efficient, accurate and reliable method for determining the melting temperature without any prior knowledge of the solid phase structure^63,64^. Because it explores the entirety of configurational space including gas, liquid and solid phases, NS also serves a test for model robustness. This robustness is partly achieved by the addition of the auxiliary repulsive pair potential, *V*_2_, an example of which is shown in Fig. 6. Core repulsion below spline point *r*_*p*_ is ensured by the addition of a repulsive core shaped *r*^−1^*e*^−*αr*^, where *α* is a tuned such that the derivatives across the spline point are smooth^60^.

The NS simulations were carried out using 896 walkers and 32 atom unit cells (29 Al and 3 Si) using the PYMATNEST software^65^. The NS walkers were moved using 1024 steps per NS iteration, each step consisting of MD to move atoms (using a 0.1 fs timestep) and MC for unit cell volume, shearing, stretching and atom-swapping steps, in a ratio of 6:6:6:6, respectively. The pressure was set to 0.1 GPa and the minimum aspect ratio of the unit cell was set to 0.85.

**Fig. 7:**
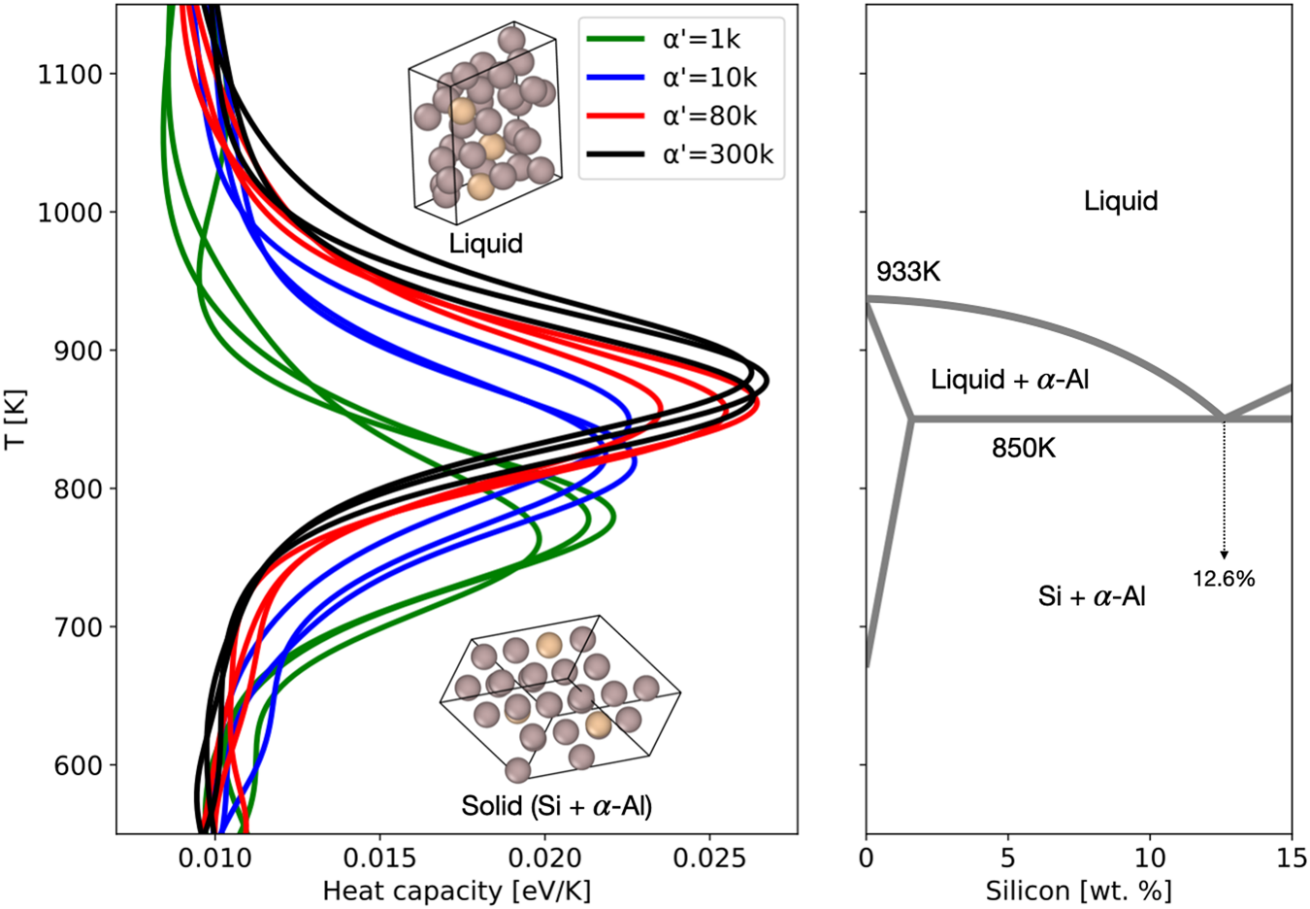
NS determined heat capacity *C*_*P*_ for ARD fitted linear AlSi10 ACE models (left) and schematic phase diagram for AlSi10^75^ (right). Increasing melting temperature accuracy is demonstrated for fits with large ${\alpha }^{{\prime} }$.

Three independent NS simulations were performed for each of the ACE models fitted to the AlSi10 HAL database and the corresponding heat capacity curves shown in Fig. 7. All models predicted the expected fcc ground structure, as confirmed using OVITO’s^66^ common neighbour analysis, but a difference in the predicted melting temperature for varying ${\alpha }^{{\prime} }$ can be seen. Only the ${\alpha }^{{\prime} }=300{\rm{k}}$ and ${\alpha }^{{\prime} }=80{\rm{k}}$ models accurately predict the melting temperature of 867 K as given by Thermo-Calc with the TCAL4 database^67^. Comparison with Table 2 suggests that a test accuracy of at least 5 meV/atom is required to determine the melting temperature accurately.

### Polyethylene glycol (PEG)

This section presents the application of HAL to build databases for polymers. Polyethylene glycol (PEG) has the formula H[OCH_2_CH_2_]_n_OH, where *n* is the number of monomer units^68^. From a modelling perspective these polymers are challenging to simulate in vacuum as they form configurations ranging from tightly coiled up to fully stretched out structures. Due to the OH group at the end the polymer can also exhibit hydrogen bonding, which further complicates its description. These hydrogen bonds typically correspond to low-energy configurations and are frequently formed and broken during long MD simulations. This section first presents a benchmark of HAL against AL followed by a demonstration HAL finding configurations exhibiting large errors. Finally, the potential fitted to small polymer units in vacuum is used to predict the density of a long PEG(*n* = 200) polymer in bulk with good accuracy relative to experiment. All DFT reference calculations in this section are carried out with the ORCA code^69^ using the *ω*B97X DFT exchange correlation functional^70^ and 6-31G(d) basis set.

In order to test whether HAL accelerates training database assembly relative to standard AL, a benchmark test was performed. An initial database containing 20 PEG(*n* = 2) polymer configurations was created by running 500 K NVT molecular dynamics simulation using the general purpose ANI-2x forcefield^11^ sampling structures after every 7000 steps (7 ps) to provide training and test configurations to be used in the following subsections. These ANI-2x sampled configurations were then evaluated using the ORCA DFT using the parameters outlined in the previous paragraph.

This database was fitted using an ACE basis containing basis functions up to correlation order *ν* = 3 and polynomial degree 10 with an outer cutoff 4.5 Å and inner cutoff 0.5 Å. The auxiliary pair potential basis up to polynomial degree 10 and outer cutoff 5.5 Å and did not have an inner cutoff. The weights for the energy *w*_*E*_, forces *w*_*F*_ were set to 15.0 and 1.0 and remain constant throughout this section on PEG. AL (non-biasing, or *τ* = 0.0) and HAL simulations with varying biasing strengths *τ*_r_ were performed using a timestep of 0.5 fs at 500 K. Configurations were evaluated using ORCA DFT once *s*^tol^ = 0.5 was reached.

**Fig. 8:**
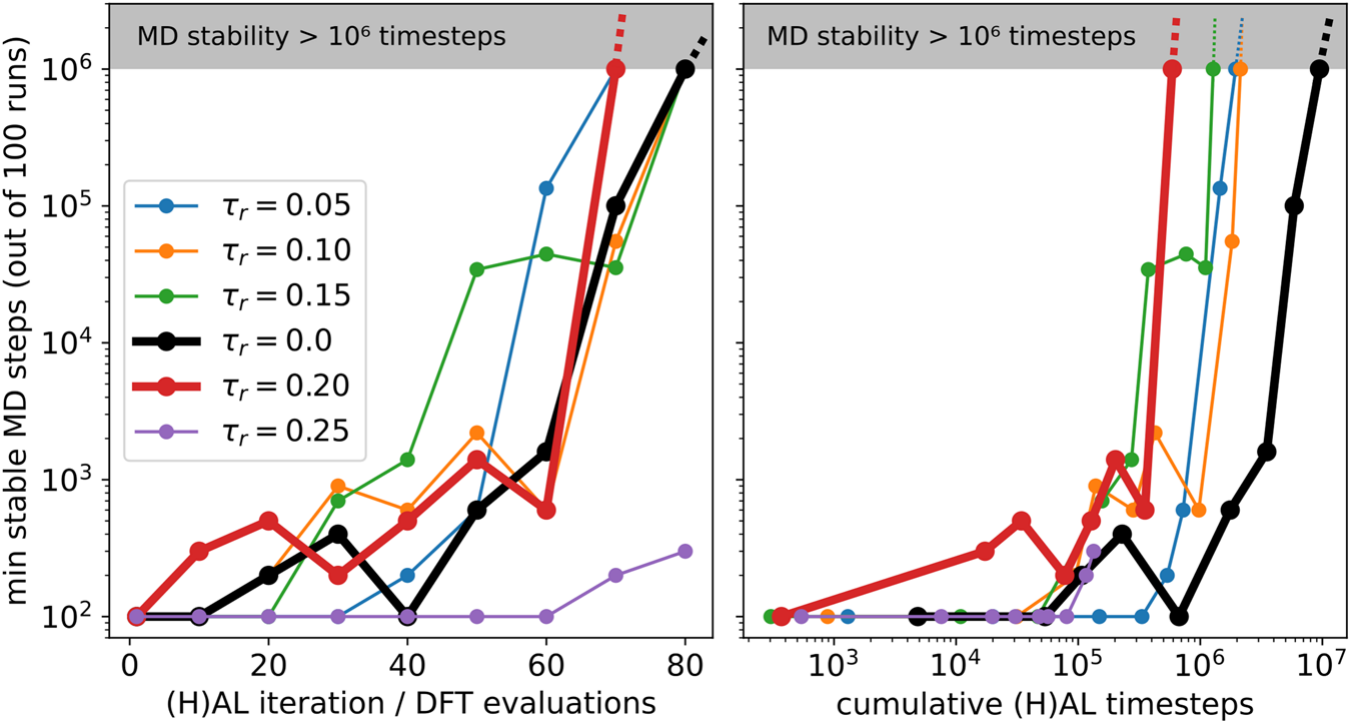
HAL vs. AL benchmark comparing MD stability for one million MD steps over 100 seeds. Turning on biasing (non-zero *τ*_r_) creates ACE models achieving stable 100 million MD timestep faster than standard AL by up to an order of magnitude.

The linear ACE models generated during the AL/HAL simulations were saved and subsequently used in a regular MD stability test and ran for 1 million MD steps at 500 K using a 1 fs timestep for 100 separate runs. A MD simulation was deemed stable if the CC and CO bonds along the chain where within 1.0–2.0 Å and the CH and OH bonds within 0.8–2.0 Å during the simulation. The minimum number of stable MD timesteps out of the 100 different simulations is shown in Fig. 8 and demonstrates that up to *τ*_r_ = 0.20 a total of 80 (H)AL iterations are required in order to achieve a minimum MD stability of 1 million steps. The large biasing strength of *τ*_r_ = 0.25 results in unstable MD dynamics as too strong biasing causes the generation of exceedingly high energy configuration far away from the desired potential energy surface to be included in the training database. Fitting to these configurations leads to a poorly performing model as many unphysical configurations enter the training database resulting.

The HAL run using a biasing strength of *τ*_r_ = 0.20, achieves minimum 1 million step MD stability after an order of magnitude fewer exploratory MD timesteps compared to standard AL. This demonstrates that HAL can be used to significantly reduce simulation time required to generate a stable potential, even though a similar amount of training configurations may be required as in a standard AL approach.

Using PEG(*n* = 4) polymers this section will investigate the ability of HAL to generate and detect configurations with large errors. First a training database was built using the general purpose ANI-2x forcefield^11^ at 500 K and 800 K using a timestep of 1 fs. Configurations were sampled every 7000 timesteps (7 ps), and used to assemble 500 K and 800 K databases. The 500K database was divided into 750 train configurations and 250 test configurations. The 800 K training and test databases both contained 250 configurations. The linear ACE model was extended to include basis functions up to 12 for both the ACE and pair potential, while keeping the cutoffs and correlation order the same (*ν* = 3) too compared to the previous section on PEG(*n* = 2).

**Fig. 9:**
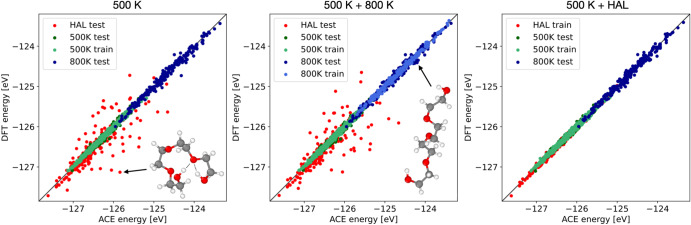
Energy scatter plots for the 500 K (left), 500 K + 800 K (middle) and 500 K + HAL (right) ACE models. HAL configuration mostly exhibit (double) hydrogen bonding, or rare events, not contained in the MD 500 K/800 K decorrelated samples.

Using the 500 K MD sampled training database HAL was started using *τ*_r_ = 0.10 and a timestep of 0.5 fs. The stopping criterion *s*^tol^ set to 0.5. A total of 200 HAL configurations were generated and formed a HAL database used for both a train and test set. Using the previously described basis three models were created fitted to: 500 K, 500 K + 800 K and a 500 K + HAL. Energy scatter plots for these three models are shown in Fig. 9 demonstrating that the errors on the HAL-found configurations are large for both the 500 K and 500 K + 800 K fits, despite the fact that the these HAL-found configurations are also low in energy. Only by including the HAL configurations in the training database can the errors on these configurations be reduced as shown in Table 3. Inspection of the HAL generated structures exposes a shared characteristic: most of them contain (double) hydrogen bonding across the polymer an example of which is shown in Fig. 9. Such hydrogen-bond formation is a rare event in this system, because only the two ends of the molecule are capable of hydrogen bonding. It is difficult to find these configurations using regular MD (even when using elevated temperatures), whereas HAL finds them easily.

**Table 3:** Train and test errors for energies (*E*) in meV and forces (*F*) in meV/Å for the 500 K, 500 K + 800 K and 500 K + HAL databases using ACE.

	No.	500 K		500 K + 800 K		500 K + HAL	
	configs	*E*	*F*	*E*	*F*	*E*	*F*
500 K train	750	30.2	58.3	32.9	60.8	32.4	59.6
500 K test	250	49.2	79.3	48.8	76.7	41.6	71.0
800 K train	250	–	–	40.0	76.4	–	–
800 K test	250	72.7	187.2	67.6	107.7	67.9	102.6
HAL	200	310.9^a^	427.2^a^	311.9^a^	404.6^a^	47.8^b^	63.4^b^

^a^is test error.

^b^is train error.

As a final investigation the density of a PEG(*n* = 200) polymer containing 1400 atoms is determined using an ACE model fitted to a HAL generated PEG training database containing polymer sizes ranging from *n* = 2 to *n* = 32 monomer units. This database contained configurations from the previous PEG sections and extended using configurations sized *n* = 8, *n* = 16 and *n* = 32. The training database included standard ANI MD sampled configurations at 500K including 1000 PEG(*n* = 4) configurations (from the previous section), as well as 50 PEG(*n* = 2), 100 PEG(*n* = 8), 100 PEG(*n* = 16) and 18 PEG(*n* = 32) configurations. Starting from this data HAL was used to generate an extra 64 PEG(*n* = 16) and 91 PEG(*n* = 32) HAL configurations until dynamics was deemed stable. The linear ACE basis used for the regression task was identical to the ACE in the previous section on PEG(*n* = 4), and any force components with greater than 20 eV/Å were excluded from the fit in order to prevent fitting on forces too far away from equilibrium.

Using the ACE model a PEG(*n* = 200) polymer was simulated in LAMMPS^71^ with the PACE evaluator pair style with periodic boundary conditions. Since the training database only contained small polymers segments in vacuum this periodic simulation demonstrates a large degree of extrapolation to configurations far away from the training database. Furthermore, the DFT code used to evaluate the training energies and forces does not support periodic boundary conditions making DFT simulation of the 1400 atom PEG(*n* = 200) simulation box not just computationally infeasible, but practically impossible in this case.

**Fig. 10:**
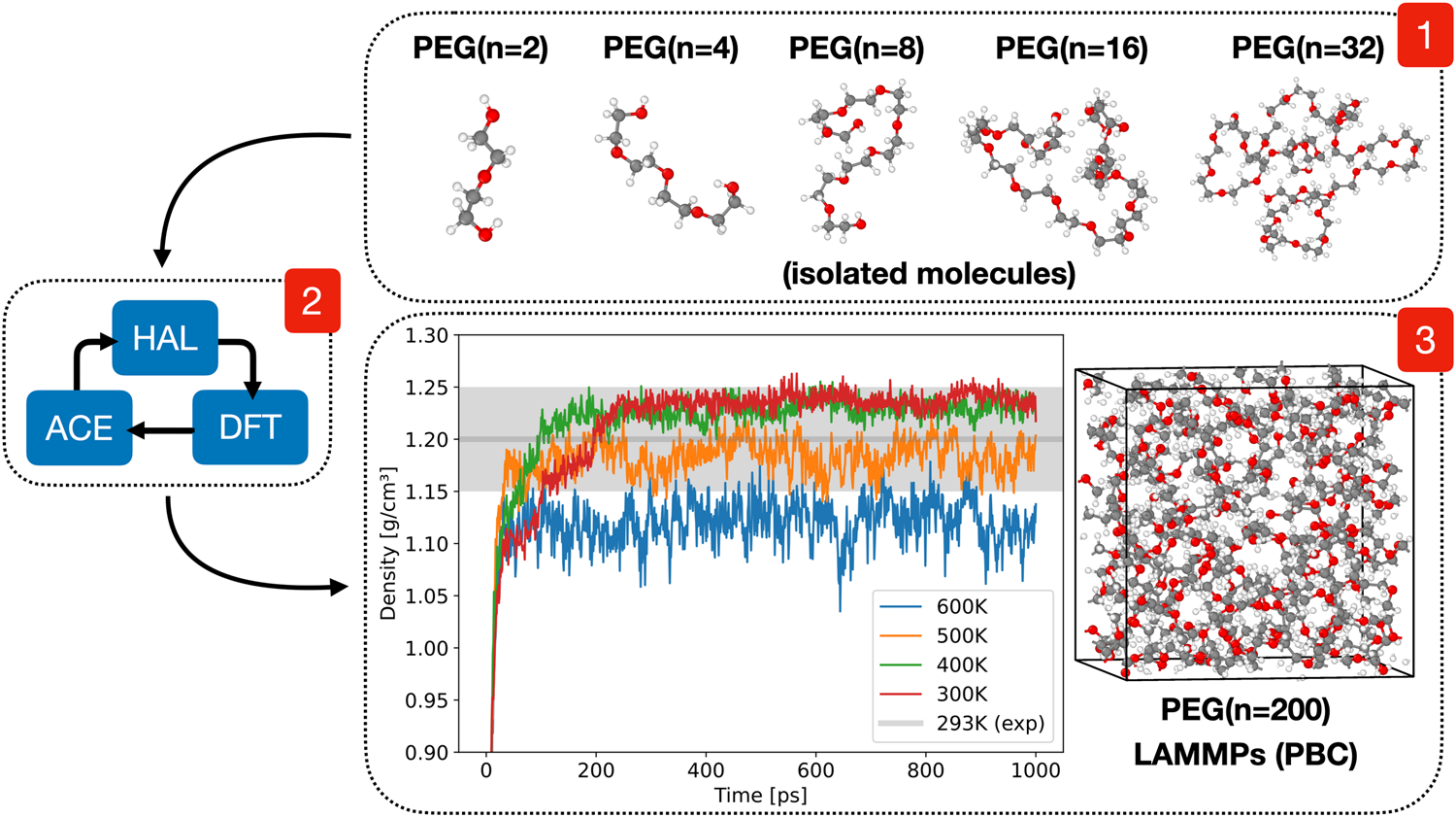
HAL protocol for building linear ACE PEG model accurately determining PEG(*n* = 200) density within experimental accuracy of 1.2 g/cm^3^ at 297 K (shaded area)^72^. Training database only included small polymers ranging from *n* = 2 to *n* = 32 in isolation.

The resulting linear ACE model was timed at 220 core-μs/atom per MD step. LAMMPs NPT simulations were performed at 1 bar using a 1 fs timestep at 300 K, 400 K, 500 K and 600 K. The recorded density as a function of simulation time is plotted in Fig. 10. Using the last 500 ps from the 300 K simulation the density was determined to be 1.238 g/cm^3^. This value is around 3% higher than the experimental value of 1.2 g/cm^3^^72^.

## Methods

### Hyperactive learning (HAL)

The HAL potential energy *E*_HAL_ as defined in Eq. (1) biases MD simulations during the exploration step in AL towards uncertainty by shifting the potential energy surface and assigning lower energies to configurations with high uncertainty. Considering $\tilde{\sigma }$ defined in Eq. (5), its gradient $\nabla \tilde{\sigma }$ can be computed as:

11 $$\nabla \tilde{\sigma }=\frac{\nabla {\tilde{\sigma }}^{2}}{2\tilde{\sigma }}$$

where

12 $$\begin{array}{lll}\nabla {\tilde{\sigma }}^{2}\,=\,\frac{2}{K}\mathop{\sum }\limits_{k=1}^{K}\left({E}^{k}-\bar{E}\right)\left(\nabla {E}^{k}-\nabla \bar{E}\right)\\ \qquad\,\,=\,\frac{2}{K}\mathop{\sum }\limits_{k=1}^{K}\left({E}^{k}-\bar{E}\right)\left(\bar{F}-{F}^{k}\right)\end{array}$$

and *F*^*k*^ = − ∇ *E*^*k*^, $\bar{F}=-\nabla \bar{E}$. These predictions are obtained by parameterisations ${\{{{{{\bf{c}}}}}_{k}\}}_{k = 1}^{K}$, while $\bar{{{{\bf{c}}}}}$ is the analytic mean of the posterior distribution as specified in Eq. (25). The *K*-sum runs over the energy and force predictions from the committee models. Other architectures such as neural networks ensembles may be considered in future work. This quantity in essence is a computationally cheap method of determining the gradient towards (total) energy uncertainty and may be interpreted as a conservative biasing force:

13 $${F}^{\tilde{\sigma }}:= \nabla \tilde{\sigma }.$$

HAL dynamics adds this biasing force to MD in order to accelerate the generation of configurations with high uncertainty, which sets HAL apart from AL. Setting *τ* = 0 recovers standard MD dynamics, and in this sense, HAL generalises AL. Interestingly, previous work employed a biasing force using a neural network interatomic potential^73^ but biased away from uncertainty in order to stabilise the MD dynamics.

The biasing strength *τ* can either be set as a constant or adapted during the HAL simulation. Controlling the biasing strength is important as too strong biasing can quickly lead to unphysical configurations, whereas low biasing generates valuable configurations at a slow rate. The adaptive biasing works by first setting *τ*_r_ and performing a burn-in period to record the magnitudes (or, norms) of ${F}^{\tilde{\sigma }}$ and $\bar{F}$. Typically, the burn-in period is set to the history of the latest 100 timesteps *δt* to estimate the degree of uncertainty (or extrapolation) and adjust the biasing strength accordingly. The biasing strength *τ* is given by:

14 $$\tau =\frac{{\tau }_{{{{\rm{r}}}}}\mathop{\sum }\nolimits_{m = 1}^{100}\parallel \bar{F}(t-m\delta t)\parallel }{\mathop{\sum }\nolimits_{m = 1}^{100}\parallel {F}^{\tilde{\sigma }}(t-m\delta t)\parallel },$$

where the relative biasing parameter *τ*_r_ is generally set in the range 0.05 to 0.20 (see Fig. 8 for a numerical study). It can be understood as the approximate relative average strength of the biasing force in comparison to the average force of the fitted model. Using this adaptive biasing term aids usability and tunes the biasing strength to ensure that HAL gently drives MD towards high uncertainty. The value may loosely be interpreted as the relative magnitude of the biasing force compared to the true gradient of the potential energy surface. Larger *τ*_r_ increases the biasing strength and rate at which configurations with high uncertainty are generated. In order to sample configurations at desired pressures and temperatures a proportional control barostat was added as well as a Langevin thermostat.

### Atomic cluster expansion (ACE)

The ACE model decomposes the total energy *E* of a configuration *R* as a sum of parameterised atomic energies:

15 $$E({{{\bf{c}}}};R)=\mathop{\sum}\limits_{i\in R}{E}_{i}({{{\bf{c}}}};R).$$

The atomic energies *E*_*i*_ are linear combinations of ACE basis functions, i.e., *E*_*i*_(**c**; *R*) = **c** ⋅ ${\boldsymbol{B}}_i$(*R*). Here, ${\boldsymbol{B}}_i$(*R*) denotes the evaluation of the ACE basis on the atomic site environment of the *i*th atom, ${\{({{{{\boldsymbol{r}}}}}_{ij},{z}_{j})\}}_{j}$, which consists of relative positions ***r***_*ij*_ = ***r***_*j*_ − ***r***_*i*_ and associated chemical elements *z*_*j*_, denoted by the atomic number, of neighbouring atoms *j*. In this work it is chosen to project the atomic site environment onto the following single-element basis function *ϕ*_*znlm*_:

16 $${\phi }_{znlm}({{{{\boldsymbol{r}}}}}_{ij},{z}_{j})={\delta }_{z{z}_{j}}{R}_{n}({r}_{ij}){Y}_{lm}({\hat{{{{\boldsymbol{r}}}}}}_{ij}),$$

followed by a pooling operation resulting in features:

17 $${A}_{iznlm}=\mathop{\sum}\limits_{j}{\phi }_{znlm}({{{{\boldsymbol{r}}}}}_{ij},{z}_{j}),$$

that are denoted the atomic basis in the context of the ACE model. Taking a *ν* order (tensor) product results in many-body correlation functions incorporating (*ν* + 1) body-order interactions:

18 $${{{{\boldsymbol{A}}}}}_{i{{{\bf{znlm}}}}}=\mathop{\prod }\limits_{t=1}^{\nu }{A}_{i{z}_{t}{n}_{t}{l}_{t}{m}_{t}}.$$

The **A**-basis is a complete basis of permutation-invariant functions but does not incorporate rotation or reflection symmetry. An isometry invariant basis ${\boldsymbol{B}}$ is constructed by averaging over rotations and reflections. Representation theory of the orthogonal group *O*(3) shows that this can be expressed as a sparse linear operation and results in:

19 $${{{{\boldsymbol{B}}}}}_{i}={{{\boldsymbol{C}}}}{{{{\boldsymbol{A}}}}}_{i},$$

where ***C*** contains generalised Clebsch-Gordan coefficients; we refer to^22,23^ for further details.

A major benefit of the linear ACE model is that the computational cost of evaluating a site energy *E*_*i*_ scales only linearly with the number of neighbouring atoms, as well as with the body order *ν* + 1.

### (Bayesian) Linear regression

The parameters of linear ACE models are fitted by solving a linear regression problem. The associated squared loss function *L*(**c**) to be minimised over configurations *R* in training set ${\boldsymbol{R}}$ with corresponding (DFT) observations for energy ${{{{\mathcal{E}}}}}_{R}$, forces ${{{{\mathcal{F}}}}}_{R}$ is:

20 $$\begin{array}{r}L({{{\bf{c}}}})=\mathop{\sum}\limits_{R\in {{{\boldsymbol{R}}}}}{{w}_{E}}^{2}| E({{{\bf{c}}}};R)-{{{{\mathcal{E}}}}}_{R}{| }^{2}+\\ +{{w}_{F}}^{2}| F({{{\bf{c}}}};R)-{{{{\mathcal{F}}}}}_{R}{| }^{2}\end{array}$$

where *w*_*E*_ and *w*_*F*_ are weights specifying the relative importance of the DFT observations. When fitting materials a third term is added ${{w}_{V}}^{2}| V({{{\bf{c}}}};R)-{{{{\mathcal{V}}}}}_{R}{| }^{2}$ referring to the virial stress components of the configuration *R*. This minimisation problem can be recast in the generic form:

21 $$\arg \mathop{\min }\limits_{{{{\bf{c}}}}}\,\,\parallel {{{\bf{y}}}}-{{{\mathbf{\Psi }}}}{{{\bf{c}}}}{\parallel }^{2}+\eta \parallel {{{\bf{c}}}}{\parallel }^{2},$$

where ${{{\mathbf{\Psi }}}}\in {{\mathbb{R}}}^{{N}_{{{{\rm{obs}}}}}\times {N}_{{{{\rm{basis}}}}}}$ is the design matrix and the observation vectory $\in {{\mathbb{R}}}^{{N}_{{{{\rm{obs}}}}}}$ collects the observations to which the parameters are fitted. Entries in the design matrix and the observations vector corresponding to force observations and observations of virials are scaled by a factor of *w*_*E*_/*w*_*F*_ and *w*_*V*_/*w*_*F*_, respectively, to account for the relative weighting of the penalty terms in (20). Here, we also added a Tychonov regularisation with regularisation parameter *η* > 0 which is commonly determined through a model selection criterion such as cross-validation.

This linear regression model can be cast in a Bayesian framework by specifying a prior distribution *p*(**c**) over the regression parameters, and an (additive) probabilistic error models ${\epsilon }_{R}^{E},{\epsilon }_{R}^{F}$ which give rise to the generative model:

22 $$\begin{array}{l}{{{{\mathcal{E}}}}}_{R}=E({{{\bf{c}}}};R)+{\epsilon }_{R}^{E},\\ {{{{\mathcal{F}}}}}_{R}=F({{{\bf{c}}}};R)+{\epsilon }_{R}^{F},\end{array}$$

for *R* ∈ ${\boldsymbol{R}}$. This generative model can be written in short-hand form as:

23 $${{{\bf{y}}}}={{{\mathbf{\Psi }}}}{{{\bf{c}}}}+{{{\boldsymbol{\epsilon }}}},$$

where ***ϵ*** is a linear transformation of the error models ${\epsilon }_{R}^{E},{\epsilon }_{R}^{F}$, *R* ∈ ${\boldsymbol{R}}$.

In the context of this work, ${\epsilon }_{R}^{E},{\epsilon }_{R}^{F}$ model random perturbations of DFT calculations and are assumed to be mainly present due to the locality assumption and DFT convergence properties, e.g. k-point sampling. For simplicity we assume in this work that the entries of the error model **ϵ** in the generic representation (21) are statistically independent and Gaussian distributed with mean 0 and precision (inverse variance) *λ*. In terms of the model (22) this assumption implies ${\epsilon }_{R}^{E} \sim {{{\mathcal{N}}}}(0,{\lambda }^{-1})$, ${\epsilon }_{R}^{F} \sim {{{\mathcal{N}}}}({{{\bf{0}}}},{{{\bf{I}}}}{w}_{E}^{-2}{w}_{F}^{2}{\lambda }^{-1})$. In principle, extension to other noise models can be made.

The here assumed noise model gives rise to the likelihood function:

24 $$p({{{\bf{y}}}}| {{{\boldsymbol{R}}}},{{{\bf{c}}}},\lambda )={\left(\frac{\lambda }{2\pi }\right)}^{{N}_{{{{\rm{obs}}}}}/2}\exp \left\{-\frac{\lambda }{2}\parallel {{{\bf{y}}}}-{{{\mathbf{\Psi }}}}{{{\bf{c}}}}{\parallel }^{2}\right\}$$

By restricting ourselves to a Gaussian error model, and assuming the prior to be Gaussian as well, i.e., $p({{{\bf{c}}}})={{{\mathcal{N}}}}({{{\bf{c}}}}| {{{\bf{0}}}},{{{{\mathbf{\Sigma }}}}}_{0})$, it is ensured that the posterior distribution, *π*(**c**) = *p*(**c**∣${\boldsymbol{R}}$, **y**, *λ*), is Gaussian with closed form expressions for both the distribution mean $\bar{{{{\bf{c}}}}}$ and variance **Σ**:

25 $$\begin{array}{lll}\quad\,\,\bar{\bf{c}}\,=\,\lambda {\mathbf{\Sigma }}{{{{\mathbf{\Psi }}}}}^{T}{{{\bf{y}}}}\\ \mathop{\mathbf{\Sigma}}\nolimits^{-1}\,=\,\mathop{\mathbf{\Sigma}}\nolimits_{0}^{-1}+\lambda {{{{\mathbf{\Psi }}}}}^{T}{{{\mathbf{\Psi }}}}.\end{array}$$

In the context of this work, having closed form expressions for both these quantities is desirable as it (1) allows for conceptual easy and fast generation of independent samples ${\{{{{{\bf{c}}}}}^{k}\}}_{k = 1}^{K}$ from the posterior distribution, and (2) allows for a parametrisation of the fitted model with the exact mean, $\bar{{{{\bf{c}}}}}$, of the posterior distribution.

In what follows we briefly describe two Bayesian regression techniques, Bayesian ridge regression (BRR), which we use to produce Bayesian fits during the HAL data generation phase, and the computationally more costly automatic relevance determination (ARD), which we typically use to obtain a final model fit after the data generation is complete.

### Bayesian ridge regression (BRR)

In Bayesian ridge regression the covariance of the prior is assumed to be isotropic, i.e.:

26 $$p({{{\bf{c}}}}| \alpha )={{{\mathcal{N}}}}({{{\bf{c}}}}| {{{\bf{0}}}},{\alpha }^{-1}{{{\bf{I}}}}),$$

for some hyperparameter *α* > 0, the precision of the prior distribution.

Under this choice of prior, the logarithm of the posterior distribution takes the form:

27 $$\ln \pi ({{{\bf{c}}}})=-\frac{\lambda }{2}\parallel {{{\bf{y}}}}-{{{\mathbf{\Psi }}}}{{{\bf{c}}}}{\parallel }^{2}-\frac{\alpha }{2}\parallel {{{\bf{c}}}}{\parallel }^{2}+{{{\rm{C}}}},$$

where *C* is some constant. Thus, maximising the (log-)posterior for this choice of prior, is equivalent to solving the regularised least square problem Eq. (27) with ridge penalty *η* = *λ*/*α*. This shows that the prior naturally gives rise to a regularised solution, keeping coefficient parameters small.

The determination of the hyperparameters *α* and *λ* in BRR is achieved by optimising the marginal log likelihood also known as evidence maximisation^74^. One first defines the evidence function as:

28 $$p({{{\bf{y}}}}| \alpha ,\lambda )=\int\,p({{{\bf{y}}}}| {{{\bf{c}}}},\lambda )p({{{\bf{c}}}}| \alpha )d{{{\bf{c}}}}$$

which marginalises out the coefficients **c** and describes the likelihood of observing the data given the hyperparameters *α* and *λ*. Using the previously defined definitions the evidence function can be expressed as:

29 $$\begin{array}{l}p({{{\bf{y}}}}| \alpha ,\lambda )={\left(\frac{\lambda }{2\pi }\right)}^{{N}_{{{{\rm{obs}}}}}/2}{\left(\frac{\alpha }{2\pi }\right)}^{{N}_{{{{\rm{basis}}}}}/2}\\ \displaystyle\int\exp \left\{-\frac{\lambda }{2}\parallel {{{\bf{y}}}}-{{{\mathbf{\Psi }}}}{{{\bf{c}}}}{\parallel }^{2}-\frac{\alpha }{2}\parallel {{{\bf{c}}}}{\parallel }^{2}\right\}\end{array}$$

where *N*_basis_ is the dimensionality of **c**. Completing the square in the exponent and taking the log gives rise to the marginal log likelihood:

30 $$\begin{array}{ll}\ln p({{{\bf{y}}}}| \alpha ,\lambda )=\frac{{N}_{{{{\rm{basis}}}}}}{2}\ln \alpha +\frac{{N}_{{{{\rm{obs}}}}}}{2}\ln \lambda \\ \qquad\qquad\qquad-\,\frac{\lambda }{2}\parallel {{{\bf{y}}}}-{{{\mathbf{\Psi }}}}{{{\bf{c}}}}{\parallel }^{2}-\frac{\alpha }{2}\parallel {{{\bf{c}}}}{\parallel }^{2}\\ \qquad\qquad\qquad+\,\frac{1}{2}\ln \parallel {{{\mathbf{\Sigma }}}}\parallel -\frac{{N}_{{{{\rm{obs}}}}}}{2}\ln (2\pi )\end{array}$$

which can be maximised with respect to *α* and *λ* in order maximise the marginal likelihood and obtain the statistically most probably likely solution given the basis and data.

### Automatic relevance determination (ARD)

Automatic relevance determination (ARD) modifies BRR by relaxing the isotropy of the prior and assigning a hyperparameter *α*_*i*_ to independently regularise each coefficient *c*_*i*_. The corresponding prior is given by:

31 $$\begin{array}{ll}p({{{\bf{c}}}}| {{{\boldsymbol{\alpha }}}})={{{\mathcal{N}}}}({{{\bf{c}}}}| {{{\bf{0,}}}}{{{{\mathcal{A}}}}}^{-1})\\ \qquad{{{\mathcal{A}}}}={{{\rm{diag}}}}({\alpha }_{1},...,{\alpha }_{{N}_{{{{\rm{basis}}}}}}).\end{array}$$

This prior determines the relevance of each parameter *c*_*i*_, or basis function, which effectively results in a feature selection. Basis functions are ranked based on their relevance and are pruned if determined irrelevant, in turn producing a sparse solution. In practice, sparse models obtained through ARD often yield better generalisation than BRR. Using ARD requires the specification of a threshold parameter ${\alpha }^{{\prime} }$ setting the minimum relevance of basis functions included in the fit. Adjusting this parameter controls the balance between accuracy and sparsity of the model.

### Posterior predictive distribution

A key property of the Bayesian approach is that it provides a way to quantify uncertainty of model predictions in terms of the posterior-predictive distribution, which accounts both for parameter uncertainty as given by the posterior distribution as well as uncertainty due to observation error.

For example, the probabilistic description of the predicted energy E^*^ at a configuration R^*^ is:

32 $$\begin{array}{ll}{E}^{* }=E({{{\bf{c}}}};{R}^{* })+{\varepsilon }_{{R}^{* }}^{E},\\ {\varepsilon }_{{R}^{* }}^{E} \sim {{{\mathcal{N}}}}(0,{\lambda }^{-1}),\\ \quad{{{\bf{c}}}} \sim \pi ({{{\bf{c}}}}).\end{array}$$

Thus, the posterior predictive distribution of energy, i.e., the conditional distribution *p*(E^*^∣R^*^), can be verified to be normal:

33 $$\begin{array}{lll}p({E}^{* }| {R}^{* })\,=\,\displaystyle\int\,p({E}^{* }| {R}^{* },{{{\bf{c}}}})\pi ({{{\bf{c}}}})d{{{\bf{c}}}}\\ \qquad\qquad\,\,\,=\,{{{\mathcal{N}}}}({E}^{* }| \bar{{{{\bf{c}}}}}\cdot {{{\boldsymbol{B}}}},{\sigma }^{2}),\end{array}$$

where the variance *σ*^2^ is as specified in Eq. (3).

Closed forms of the predictive distribution of other quantities that are linear transformations of the coefficients **c** and the noise model can be similarly derived. For quantities that are non-linear and potentially only implicitly defined transformations, approximations of their predictive distribution can be obtained by propagation of the Monte Carlo samples {**c**_*k*_}.

## Data Availability

The code, potentials and databases used to generate these potentials can be found on the ACEHAL github page https://github.com/ACEsuit/ACEHAL.
